# Barriers and Challenges to Conducting Research Within the NHS: Reflections from Professionals Within Specialist Child and Adolescent Mental Health Services (CAMHS)

**DOI:** 10.1192/bjo.2026.11238

**Published:** 2026-06-30

**Authors:** Leah Evans, Elsie Williams, Rachel Elvins, Alison Wood

**Affiliations:** 1 Greater Manchester Mental Health NHS Trust, Bolton, United Kingdom; 2 Manchester University NHS Foundation Trust, Manchester, United Kingdom; 3 Pennine Care NHS Foundation Trust, Manchester, United Kingdom

## Abstract

**Aims:**

The Eating Disorder Online Activity Questionnaire was developed within a specialist Community Eating Disorder Service (CEDS) to explore online activity of Children and Young People presenting with eating disorders. The authors proposed a research project to evaluate its psychometric properties in adolescents receiving care from CEDS. Despite this being a low-risk observational study with no funding requirements, the research team have encountered several challenges resulting in significant delays to the planned timeline. This study aims to present practice-based reflections on the barriers to conducting low-risk research studies within the NHS.

**Methods:**

A literature search was conducted to review current evidence relevant to NHS research processes. Journal articles and policy documents were synthesised to highlight barriers and facilitators for conducting research in the NHS. Authors used Gibbs reflective cycle to reflect on key challenges which contributed to a twelve-month timeline from first developing their research proposal to sponsorship application.

Authors categorised factors which led to the delay and then used these categories to create an analysis and draw conclusions. Analysis and conclusions were linked back to the results from the literature search. Final conclusions and background literature were used to inform recommendations for research policy.

**Results:**

Delays reflect major structural challenges within NHS research processes. Research teams face several systemic challenges, even for small-scale, low-risk studies. Key issues include a lack of clear and accessible guidance, complex and inconsistent application processes, and under-resourced Research and Innovations (R&I) departments. Organisations were reluctant to agree to sponsorship of an unfunded study despite low risks associated and costs being accounted for within the practitioners training scheme. Sponsorship applications added a high administrative burden and were inconsistent across different organisations.

**Conclusion:**

Small-scale research projects are integral to the national ambition to increase research active organisations and allow early access to treatment, improve patient outcomes and increase staff satisfaction. Despite this, low risk studies face significant challenges and are routinely de-prioritised by sponsorship organisations due to lack of funding. In the case study, these delays had implications for academic progression within the team and it was reflected that these issues are likely to discourage practitioners balancing clinical and academic responsibilities from taking part in research.

Recommendations:

A standardised application process across trusts and organisations to improve transparency and reduce administrative burden on practitioners.

A simplified, streamlined process for small-scale, low-risk studies which do not present significant financial costs or ethical considerations.

